# Genomic Analysis of *Staphylococcus aureus* Isolates Associated With Peracute Non-gangrenous or Gangrenous Mastitis and Comparison With Other Mastitis-Associated *Staphylococcus aureus* Isolates

**DOI:** 10.3389/fmicb.2021.688819

**Published:** 2021-07-08

**Authors:** Silja Åvall-Jääskeläinen, Joanna Koort, Heli Simojoki, Suvi Taponen

**Affiliations:** ^1^Department of Veterinary Biosciences, Faculty of Veterinary Medicine, University of Helsinki, Helsinki, Finland; ^2^Department of Production Animal Medicine, Faculty of Veterinary Medicine, University of Helsinki, Saarentaus, Finland; ^3^Department of Agricultural Sciences, Faculty of Agriculture and Forestry, University of Helsinki, Helsinki, Finland

**Keywords:** *Staphylococcus aureus*, peracute mastitis, whole-genome sequence, virulence factor, gangrenous mastitis

## Abstract

*Staphylococcus aureus* is a highly prevalent cause of mastitis in dairy herds worldwide, capable of causing outcomes that vary from subclinical to peracute gangrenous mastitis. We performed a comparative genomic analysis between 14 isolates of *S. aureus*, originating from peracute bovine mastitis with very severe signs (9 gangrenous, 5 non-gangrenous) and six isolates originating from subclinical or clinical mastitis with mild to moderate signs, to find differences that could be associated with the clinical outcome of mastitis. Of the 296 virulence factors studied, 219 were detected in all isolates. No difference in the presence of virulence genes was detected between the peracute and control groups. None of the virulence factors were significantly associated with only a single study group. Most of the variation in virulence gene profiles existed between the clonal complexes. Our isolates belonged to five clonal complexes (CC97, CC133, CC151, CC479, and CC522), of which CC522 has previously been detected only in isolates originating from caprine and ovine mastitis, but not from bovine mastitis. For statistical analysis, we sorted the CCs into two groups. The group of CCs including CC133, CC479, and CC522 was associated with gangrenous mastitis, in contrast to the group of CCs including CC97 and CC151. The presence of virulence genes does not explain the clinical outcome of mastitis, but may be affected by allelic variation, and especially different regulation and thus expression in the virulence genes.

## Introduction

*Staphylococcus aureus (S. aureus)* is a significant cause of mastitis in ruminants such as cows, sheep, and goats worldwide (Bergonier et al., 2003; Keane, 2019; Vasileiou et al., 2019). *Staphylococcus aureus* mastitis has also been detected in other animal species, such as rabbits (Corpa et al., 2009) and dogs (Hasegawa et al., 1993), as well as in humans (Angelopoulou et al., 2018). In dairy cows, the most common type of *S. aureus* mastitis is subclinical (Koivula et al., 2007; Rainard et al., 2018a), but *S. aureus* is among the most common causes of clinical bovine mastitis as well (Koivula et al., 2007; Wellnitz and Bruckmaier, 2012). A study by Vakkamäki et al. (2017) showed that *S. aureus* causes 21% of cases of clinical mastitis in dairy herds in Finland, being the second most common mastitis pathogen after non-aureus staphylococci (NAS). The severity of clinical *S. aureus* mastitis varies from mild, expressed only by changes in milk, to peracute gangrenous mastitis causing necrosis of the affected udder quarter, severe systemic signs, and even death of the cow (Rainard et al., 2018a). Severe cases are rare, but occur occasionally in dairy cows (Matsunaga et al., 1993; Shibahara and Nakamura, 1999; Shafi et al., 2015). Severe peracute gangrenous mastitis has also been detected in other ruminants such as sheep (Vautor et al., 2009) and goats (Rainard et al., 2018b), in other animals such as rabbits (Corpa et al., 2009), and in human females (Agrawal et al., 2014).

*Staphylococcus aureus* produces a broad arsenal of virulence factors (VFs) that can be involved in various stages of mastitis pathogenesis, such as attachment and invasion of host cells, tissue degradation, evasion and modulation of host immune defenses, as well as nutrient acquisition from the host (Sutra and Poutrel, 1994; Foster et al., 2014). However, knowledge gaps still exist for several VFs described for *S. aureus* regarding their significance and role in mastitis pathogenesis. The severity of *S. aureus* mastitis may be influenced both by pathogen-related factors such as differences in the expression of virulence genes (Wolf et al., 2011), as well as by the host factors such as the immunological defense capabilities of the host and to some extent by environmental stress factors (Aitken et al., 2011). However, a deeper understanding of the mechanisms explaining the course and manifestation of mastitis remains open (Rainard et al., 2018a).

In our previous study (Åvall-Jääskeläinen et al., 2018), we compared the putative virulence gene profiles of 24 staphylococcal isolates of *S. aureus* and three NAS species. Half of the isolates in each species were isolated from clinical and the other half from subclinical bovine mastitis. We were unable to show whether the presence of any of the virulence genes identified could be associated with the clinical outcome of the mastitis, i.e., clinical or subclinical. Another comparative genomics study by Rocha et al. (2019) compared the VFs of four *S. aureus* isolates from subclinical and two *S. aureus* isolates from clinical bovine mastitis. These authors likewise found no virulence genes associated with the clinical outcome of the mastitis; however, they did determine that isolates from clinical and subclinical mastitis could be separated, based on sequence variation in a membrane-anchored lipoprotein, possibly affecting the immune system of the host. However, a genomic study by Hoekstra et al. (2020) with 276 *S. aureus* isolates from both clinical and subclinical mastitis showed that multiple genes were associated with the clinical outcome of mastitis. These genes were clustered within isolates belonging to the same clonal complex (CC). Rainard et al. (2018a) speculated that a combination of certain genes would be more essential to inducing mastitis than any single virulence gene. In sheep, some differences in the VF profiles of *S. aureus* associated with subclinical and gangrenous mastitis have been identified (Vautor et al., 2009).

We aimed to determine whether the presence or absence of genes encoding the putative VFs were associated with the clinical outcome of bovine *S. aureus* mastitis. We therefore genome-sequenced 14 *S. aureus* isolates from peracute bovine mastitis with very severe signs. For comparison, a total of six *S. aureus* isolates from subclinical or clinical mastitis with milder symptoms were genome-sequenced as well.

## Materials and Methods

### Bacterial Isolates and Growth Conditions

**FIGURE 1:**
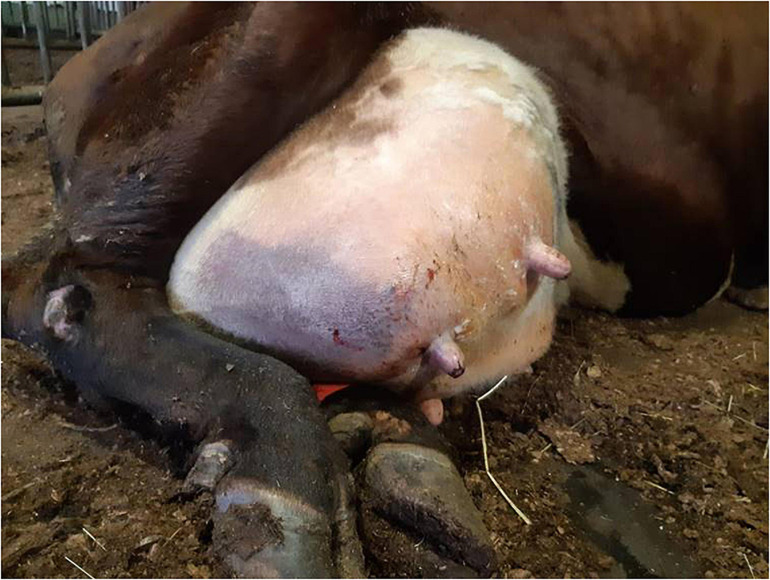
Udder of a dairy cow suffering from peracute gangrenous *Staphylococcus aureus* mastitis with ischemia and blue discoloration of the affected right hind quarter.

In the practice area of the Ambulatory Production Animal Clinic of the Faculty of Veterinary Medicine, University of Helsinki, *S. aureus* isolates from very severe bovine mastitis cases during the years 2011–2018 were collected and stored in beads (Microbank^®^, Pro-Lab Diagnostics, Neston, Cheshire, United Kingdom) at −80°C. All symptoms and other details about the mastitis cases were recorded. In all, 14 isolates, Saari 1–Saari 14, with the most severe symptoms were chosen for the study. In these mastitis cases, the cows had very severe local and systemic peracute signs. The signs included hyperthermia, depression, anorexia, recumbency, ischemia of the affected quarter causing blue discoloration on parts of the udder skin (9 cows) (Figure 1), and blood in milk (Table 1). This peracute study group was further divided into two subgroups based on the clinical signs of mastitis: peracute gangrenous group and peracute non-gangrenous group. For comparison, three *S. aureus* isolates from clinical mastitis (Saari 15, Saari 18, Saari 20) with less severe signs (changes in the milk, hard udder quarter, and increased body temperature) and three isolates from subclinical mastitis (Saari 16, Saari 17, Saari 19) from the year 2018 were stored with the study group isolates and included in the study. These six isolates are herein referred to as the controls. All 20 isolates originated from different herds in southern Finland. Eleven of the 14 study cows died or were euthanized, while information on the subsequent fate of two cows became unavailable. Four of the six control cows were culled due to *S. aureus* mastitis. One cow from the study group and two control cows survived at least 1 year after the mastitis case (Table 1). One study cow (Saari 6) was examined postmortem at Veterinary Pathology of the University of Helsinki. The postmortem examination revealed that the left intramammary lymph node was enlarged and the incision surface was suppurative with a light yellow color. The histology of the udder revealed local necrosis, and the number of neutrophils was substantially increased in the alveoli. The interstitial tissue was strongly edematous.

**TABLE 1:** *Staphylococcus aureus* isolates included in the study.

Cow and sample ID	Parity	Days in milk	Quarter^a^	Milk appearance	Quarter	Discoloration of quarter	Systemic signs	Type of mastitis	Euthanasia due to mastitis	Days alive after mastitis onset
Saari 1	3	279	LF	N/A	Hard	N/A	Yes	Severe clinical	N/A	
Saari 2	1	158	RH	Bloody	Hard	Yes	Yes	Gangrenous mastitis	Euthanized	0
Saari 3	4	7	RH	Bloody	Hard	Yes	Yes	Gangrenous mastitis	N/A	
Saari 4	6	55	LH	N/A	Hard	N/A	Yes	Severe clinical	Alive	> 365
Saari 5	N/A	7	LH	Bloody	Hard	Yes	Yes	Gangrenous mastitis	Euthanized	0
Saari 6	4	1	LF	Yellow, clots	Hard	No	Yes	Gangrenous mastitis	Euthanized	1
Saari 7	2	113	RH	Clots	Hard	No	Yes	Severe clinical	Slaughtered	119
Saari 8	3	8	RH	Clots	Hard	No	Yes	Severe clinical	Slaughtered	82
Saari 9	4	118	RF	Bloody	Hard	Yes	Yes	Gangrenous mastitis	Slaughtered	59
Saari 10	4	0	RH	Bloody	Hard	Yes	Yes	Gangrenous mastitis	Euthanized	1
Saari 11	5	5	LH	Bloody	Hard	No^b^	Yes	Gangrenous mastitis	Euthanized	31
Saari 12	2	4	RH	Bloody	Hard	Yes	Yes	Gangrenous mastitis	Euthanized	2
Saari 13	10	322	RF	Bloody	Hard	Yes	Yes	Gangrenous mastitis	Euthanized	0
Saari 14	6	79	RF	Clots	Hard	No	Yes	Severe clinical	Slaughtered	35
Saari 15	2	68	LH	Clots	Hard	No	Yes	Clinical	Alive	> 365
Saari 16	6	85	LH	Normal	Not hard	No	No	Subclinical	Slaughtered	13
Saari 17	1	173	RH	Normal	Not hard	No	No	Subclinical	Slaughtered	7
Saari 18	6	142	RH	Yellow	Hard	No	No	Clinical	Slaughtered	4
Saari 19	1	44	LH	Normal	Not hard	No	No	Subclinical	Alive	> 365
Saari 20	1	5	LH	Clots	Hard	No	Yes	Clinical	Euthanized	11

*Isolates Saari 1–Saari 14 belong to the study group and isolates Saari 15–Saari 20 to the control group. All isolates were collected from different private dairy herds in southern Finland during 2011–2018.*

*^*a*^LF, left front; LH, left hind; RF, right front; RH, right hind.*

*^*b*^Leaking of serum through the skin.*

### DNA Extraction

The isolates were cultivated aerobically on Trypticase Soy Agar supplemented with 5% bovine blood (bovine blood agar) (Tammer BioLab Oy, Tampere, Finland) at 37°C for 24 h, and the pureness of the cultures was controlled. One colony from a bovine blood agar culture of each isolate was transferred into tubes with 5 mL Müller-Hinton broth and incubated overnight at 37°C. DNA for whole-genome sequencing was extracted, using an Easy-DNA^TM^ Kit for genomic DNA isolation (Invitrogen Life Technologies, Carlsbad, CA, United States; now Applied Biosystems-Thermo Fisher Scientific Corp., Waltham, MA, United States).

### Ribotyping

Restriction endonuclease treatment of DNA was performed, using *Hin*dIII restriction enzyme (*Hin*dIII R3104L, New England BioLabs, Evry, France). Oligonucleotide probes targeting the 16S and 23S ribosomal RNA (rRNA)-encoding genes were used as described by Ragnault et al. (1997). Restriction endonuclease analysis, genomic blots, and hybridization of the membranes were performed as described by Björkroth and Korkeala (1996). The ribopatterns were scanned and imported to the BioNumerics 5.10 software package (Applied Maths, Kortrijk, Belgium). To confirm that the isolates belonged to the species *Staphylococcus aureus*, *Hin*dIII ribopatterns were compared with the corresponding patterns of 49 *Staphylococcus* type and reference strains and > 1,000 *Staphylococcus* field isolates, including 69 *Staphylococcus aureus* isolates, of our in-house ribotype library. The ribopatterns of the study isolates were compared using the Dice coefficient correlation. Unweighted Pair Group Method with Arithmetic mean (UPGMA) clustering was used for construction of the dendrogram.

### Multilocus Sequence Typing and *spa* Typing

The MLSTs and *spa* types of the isolates were analyzed. The MLSTs of the isolates were determined according to the full-length sequences of seven housekeeping genes: *arcC* (carbamate kinase), *aroE* (shikimate dehydrogenase), *glpF* (glycerol kinase), *gmk* (guanylate kinase), *pta* (phosphate acetyltransferase), *tpi* (triosephosphate isomerase), and *yqiL* (acetyl coenzyme A acetyltransferase), which were compared with reference alleles in the *S. aureus* MLST database to obtain the MLST of each isolate^1^ (Maiden et al., 2013). The MLSTs were further clustered into CCs, using eBURST version 3 (Ribeiro-Gonçalves et al., 2016). The *spa* types, based on sequences of the 24 bp repeat region of the *Staphylococcus* protein A gene *spa*, were obtained using the spaTyper version 1.0 webserver (Bartels et al., 2014) of the Center of Genomic Epidemiology^2^.

### Whole-Genome Sequencing, Assembly, Annotation, and Pangenome Construction

Genome sequencing was achieved at the Finnish Functional Genomics Center at the University of Turku and Åbo Akademi and Biocenter Finland. The quality and DNA concentrations of the extracted DNA samples were determined with Qubit^®^ fluorometric quantitation (Invitrogen Life Technologies). Sequencing libraries were prepared with TruSeq DNA polymerase chain reaction (PCR)-free LT Kit (Illumina Inc., San Diego, CA, United States). Paired-end sequencing (2 × 300 bp) was performed on the Illumina MiSeq platform (Illumina). Quality and adaptor trimming were carried out using TrimGalore (version 0.6.4) (Krueger, 2014) in paired-end mode using Cut-adapt (version 2.4) (Martin, 2011). The Phred score cutoff of 20 was used, and all reads over 20 bp long containing less than 10.0% error were retained. The quality of the RNA-seq reads and the trimmed reads was inspected using FastQC software (version 0.11.8) (Andrews, 2018).

The genomes were assembled so that the input parameters for SPAdes were first optimized, using Unicycler (version 0.4.8) (Wick et al., 2017), after which the final assembly was performed with St. Petersburg genome assembler (SPAdes) version 3.13.0 (Bankevich et al., 2012). QUAST (Quality Assessment Tool for Genome Assemblies) version 5.0.2 (Gurevich et al., 2013) was used to evaluate the quality of the assemblies. The reference genome-dependent quality metrics calculated by QUAST were obtained by comparing the genome assemblies with a previously published genome assembly for *Staphylococcus aureus* that had been retrieved from the National Center for Biotechnology Information (NCBI) database (BioProject: PRJNA231221, BioSample: SAMN11056488). Moreover, QUAST was used to evaluate the number of correctly assembled conserved genes obtained from the BUSCO (Benchmarking Universal Single-Copy Orthologs) database (Simão et al., 2015). As an additional quality metric, the percentage of reads that could be mapped to assembled genomes was evaluated. To this end, the reads were aligned using a short-read Burrows-Wheeler Aligner (BWA) (version 0.7.17) (Li and Durbin, 2010). The genome assemblies were annotated using Prokka (version 1.14.1) (Seemann, 2014) for the prediction of coding sequences (CDSs).

The pangenome was constructed using Roary version 3.11.2 with a cutoff value of 95% sequence identity (Page et al., 2015) on three different settings: (a) for the combined set of peracute and control isolates, (b) for the peracute isolates only, and (c) for the control isolates only. The piecharts visualizing the distribution of core (present in 99–100% of the isolates), soft-core (present in 95–99%), shell (present in 15% ≤ strains < 95%) and cloud (present in 0% ≤ strains < 15%) genes in all the 20 isolates were prepared using a Python script provided by Roary.

### Phylogenetic Analysis

Based on the SNP (single-nucleotide polymorphism) data of the core genes found in the pangenome analysis of both peracute and controls, a phylogenetic tree was constructed using FastTree (version 2.1.11) (Price et al., 2010). It was visualized, together with the occurrence of orthologous genes among the peracute and control samples, using a Python script provided by Roary.

### Identification of Putative Virulence Factors in the Genomes

The putative VFs sought in the genomes were chosen using the Virulence Factor Database (Liu et al., 2019), Uniprot (UniProt database)^3^, and the most recent research on the putative VFs of *S. aureus* focusing on bovine-related research, including Kane et al. (2018); Naushad et al. (2019); Rocha et al. (2019), and Hoekstra et al. (2020). In all, 296 genes encoding putative VFs, divided into six functional categories (toxins, host immune evasion, exoenzymes, adherence, secretion systems, and regulation) and a group of miscellaneous genes, were sought in the genomes of the *S. aureus* isolates. VFs were sought from the Roary-annotated pangenome sequences, using both protein and gene names, including the known synonyms as keywords when conducting the searches. Later, all the missing putative VF genes were manually sought in genomes by NCBI Basic Local Alignment Search Tool (BLAST) (megablast with default settings), using relevant reference sequences (Supplementary Table 1) from Uniprot or AureoWiki^4^ as a query. Additionally, some reference sequences were chosen from recent *S. aureus* research articles (Wilson et al., 2018; Aung et al., 2020; Hoekstra et al., 2020). The cutoff values used were: > 87% sequence identity, query coverage >52%.

All the putative VFs showing discrepancies, either as interisolate differences in the annotated names of sequences with a close identity in the pangenome comparison or as intraisolate uncertainty with sequence acquiring above cutoff hits with several VFs in the Megablast analysis, were manually checked. The protein sequences of such factors were aligned and analyzed in detail with NCBI BLAST (blastp with default settings) and, when available, compared with reviewed Uniprot (UniProt database; see text footnote 3) or other relevant reference sequences. The cutoff value used was the same as that used in the automatic annotation (*e*-value: ≤ 10^–6^). Multiple protein sequence alignments were performed with the CLUSTAL omega program (Sievers et al., 2011) when needed.

The autoinducing peptides (AIP) are encoded by the genes a*grA, agrB, agrC*, and a*grD* and classified in four Agr classes (I–IV) by the variable sequence in a*grD* encoding the autoinducing peptide (Jarraud et al., 2000). For *agrD* and *rot* (repressor of toxins) genes, further sequence analysis with NCBI megablast and CLUSTAL Omega were performed to determine the sequence variation among these genes.

### Identification of Antibiotic Resistance Genes

The genes coding for antibiotic resistance were sought in the genomes of the *S. aureus* isolates by the Resistance Gene Identifier (RGI) in the Comprehensive Antibiotic Resistance Database^5^, using default settings, but including ≥ 95% identity hits.

### Association Between the Presence of Virulence Genes and Type of Mastitis

Statistical analyses were performed in Stata MP version 16.0 (StataCorp LLC, College Station, TX, United States). The *p* < 0.05 were regarded as statistically significant. Fisher’s exact test was used to analyze the associations possible between the presence of each of the 42 virulence genes with variable presence and the type of mastitis. At first, the type of mastitis was divided into two groups (the study and control groups) and then into three groups: control group, peracute non-gangrenous, and peracute gangrenous. All virulence genes with a *p* < 0.25 [*sdrD, lukM, lukF′, ssl6, ssl7, ssl8, fhuD1, sasD, cna*, and the capsule type (5 or 8)] were retained for further analysis and seven groups of isolates were constructed, based on gene profiles. Smaller groups were combined, taking into consideration the type of mastitis: the gene profiles with 67–100% of isolates belonging to the peracute gangrenous mastitis group formed one group and the remaining gene profiles formed the other. These two groups were detected following the CCs: the group with isolates mainly from peracute gangrenous mastitis (group 2) included all isolates in CC133, CC522, and CC479, and the other group (group 1) included all isolates in CC97 and CC151. The statistical significance of the association of these two CC groups with the type of mastitis was evaluated, using a multivariate mixed-effect logistic regression model with ordinary outcome. The type of mastitis (control group/peracute non-gangrenous/peracute gangrenous) was the dependent variable and the CC group the independent variable. Parity and the lactation stage of the cow were also tested in univariable analysis and variables *p* < 0.2 were included in the final model. Parity was divided into three categories: 1–2, 3–4, or ≥ 5. The lactation stage was treated as a dichotomous variable: < 100 DIM and ≥ 100 DIM (days in milk). The final model presented consisted of parity of the cow and the CC group of the isolate.

### Gene Ontology and Clusters of Orthologous Groups Term Enrichment Analysis

The genome assemblies were functionally annotated through orthology assignment by eggNog-mapper (version 2.0.1) (Huerta-Cepas et al., 2016), allowing the assignment of Clusters of Orthologous Groups (COG) and Gene Ontology (GO) terms in the genomes. The GO- and COG-term enrichment analyses were prepared for the annotated genes that were found only in isolates causing peracute bovine mastitis (present in at least one peracute isolate, but not found in any of the control isolates) in R version 3.6.1 software (R Core Team, 2018) using clusterProfiler (package version 3.12.0) (Yu et al., 2012). All annotated genes were used as the background set. The *p*-values were adjusted, using the Benjamini-Hochberg method (Benjamini and Hochberg, 1995).

## Results

### General Features

The general features of the 20 isolates included in this study are shown in Supplementary Tables 2, 3. The mean genome size of the isolates was 2.7 Mbp. The mean total guanine-cytosine (GC) content was 32.7%, and the variance across the isolates in the content was very low. The numbers of predicted CDSs varied from 2,490 to 2,621 (Supplementary Table 3). In comparison with the highly conserved CDSs from the BUSCO database, the amount of fully covered sequences by the genome assemblies was high across all isolates, ranging from 98.6 to 99.3% (data not shown). Nine of the isolates contained one CRISPR (Clustered Regularly Interspaced Short Palindromic Repeats) locus, whereas 11 isolates had no CRISPR locus (Supplementary Table 3). The amount of different RNA-encoding genes was very similar among all isolates (Supplementary Table 3).

### Pan-, Core-, and Accessory Genome Analysis of 20 Bovine Mastitis-Related *Staphylococcus aureus* Isolates

**FIGURE 2:**
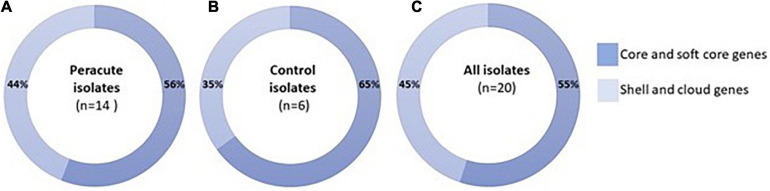
Pangenome of 20 *Staphylococcus aureus* isolates. Distribution of pangenome into core and soft-core (present in 95–100% of the isolates) and shell and cloud (present in 0% ≤ strains < 95%) genes is presented for peracute isolates **(A)**, control isolates **(B)**, and all isolates **(C)**.

As a group, the 20 *S. aureus* isolates yielded a pangenome with a size of 3,761 genes, of which 55% (2,054 genes) formed the core-genome (shared by > 99% of the isolates), revealing a high interisolate similarity (Figure 2 and Supplementary Table 4). The peracute *S. aureus* isolates yielded a pangenome with a size of 3,717 genes, which was slightly higher than the pangenome of the control *S. aureus* isolates (3,245 genes). The core-genomes of the peracute *S. aureus* isolates and control *S. aureus* isolates were 56 and 65% of the pangenome, respectively. Cloud genes (genes that are present in 0–15% of the isolates) were not observed in any of the control *S. aureus* isolates, whereas in the peracute *S. aureus* isolates they formed 23% of the pangenome (Supplementary Table 4).

**FIGURE 3:**
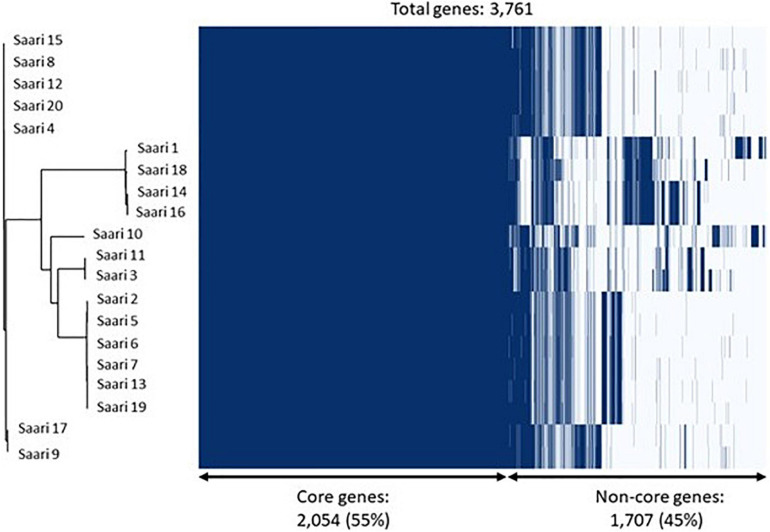
Core-genome phylogeny and gene presence across *Staphylococcus aureus* isolates. On the left, a phylogenetic tree based on the core-genomes of the 20 S. aureus isolates is shown. On the right, a heatmap showing gene presence (dark blue) or absence (light blue) of annotated genes in each of the 20 isolates. The samples have been ordered according to the phylogenetic tree.

The phylogenetic analysis of the core-genome of the 20 *S. aureus* isolates resulted in the discovery of four subgroups of isolates, all of which are mixtures of peracute and control isolates. Figure 3 combines the information on clustering of isolates into subgroups, based on the core-genome phylogenetic tree and information related to the presence and absence of annotated genes.

### Functional Classification of Coding Sequences

On average, 96.7% of the proteins deduced from the genomes of the 20 *S. aureus* isolates sequenced could be classified into COG families using eggNog-mapper. The COG family with the highest amount of proteins on average, 23%, was the category of proteins with unknown functions (Supplementary Figure 1). Among other categories with the highest abundance of proteins were proteins associated with amino-acid transport and metabolism, inorganic ion transport and metabolism, transcription as well as translation, ribosomal structure, and biogenesis (Supplementary Figure 1). The distribution of proteins in the various COG families was similar between the study groups (peracute, control). No significant differences between study groups (peracute, control) were found in the distribution of proteins in the various COG families.

The GO enrichment analysis was conducted for the gene set constructed of annotated genes found only in peracute isolates (present in at least one peracute isolate, not found in any of the control isolates) to identify overrepresented GO terms. As a result, a total of 15 significantly (adjusted *p* < 0.05) enriched GO terms, belonging to three domains (biological process, molecular function, and cellular component) were found (Table 2). Of these, five terms belonged to the biological process, nine to molecular function, and one to the cellular component domain. Several of the enriched GO terms were associated with binding to different substrates. Terms associated with transporter activity, conjugation, and aggregation were also among the enriched GO terms. No statistically significant enriched COG terms were found.

**TABLE 2:** Enriched gene ontology (GO) terms for the genes found only in the peracute samples (in at least one).

GO term	Domain	Description	*p*-value	Adjusted *p*-value
GO:0051704	Biological process	Multi-organism process	4.67E-06	0.0007744
GO:0098630	Biological process	Aggregation of unicellular organisms	3.09E-05	0.0017083
GO:0098743	Biological process	Cell aggregation	3.087E-05	0.0017083
GO:0000746	Biological process	Conjugation	0.0013566	0.0450402
GO:0009291	Biological process	Unidirectional conjugation	0.0013566	0.0450402
GO:0001968	Molecular function	Fibronectin binding	2.1E-06	8.35E-05
GO:0070051	Molecular function	Fibrinogen binding	2.1E-06	8.35E-05
GO:0044877	Molecular function	Protein-containing complex binding	7.94E-05	0.0021434
GO:0003991	Molecular function	Acetylglutamate kinase activity	0.0021786	0.0294114
GO:0015174	Molecular function	Basic amino acid transmembrane transporter activity	0.0021786	0.0294114
GO:0015181	Molecular function	Arginine transmembrane transporter activity	0.0021786	0.0294114
GO:0004721	Molecular function	Phosphoprotein phosphatase activity	0.0042846	0.0433814
GO:0034618	Molecular function	Arginine binding	0.0042846	0.0433814
GO:0005515	Molecular function	Protein binding	0.0054363	0.0489263
GO:0060187	Cellular component	Cell pole	0.0004549	0.0172876

*The terms belong to three different GO domains (biological process, molecular function, and cellular component).*

### Multilocus Sequence Types, Clonal Complexes, *spa* Types, and Ribotypes

**FIGURE 4:**
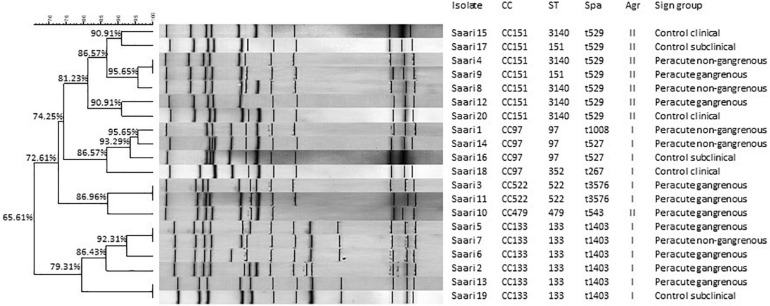
Ribotypes, clonal complexes (CC), sequence types (ST), staphylococcal protein A types (spa), and accessory gene regulator (agr) classes of 20 *Staphylococcus aureus* isolates. The ribopatterns were compared, using Dice coefficient correlation. Unweighted Pair Group Method with Arithmetic mean (UPGMA) clustering was used for construction of the dendrogram.

The MLST analysis grouped the 20 isolates into seven sequence types that belonged to five CCs: CC151 (7 isolates), CC133 (6 isolates), CC97 (4 isolates), CC522 (2 isolates), and CC479 (1 isolate) (Figure 4). Seven different spa types were detected (Figure 4). Ribotyping divided the isolates into 16 clearly distinct ribopatterns (Figure 4). Four ribopatterns included two identical isolates; in three of these ribopatterns, both isolates belonged to the peracute group, while one ribopattern included an isolate from both the peracute and control groups. The ribopatterns were further organized into four clusters with 79.31–86.96% similarity (Figure 4). These four clusters followed the CC division, except one cluster, which included both the CC479 and CC522 isolates.

### Distribution of Virulence Genes

Of the 296 VFs studied, 219 were detected in all isolates and were thus determined as core VFs. In all, 35 genes were absent from all isolates, while 42 were variably present (Supplementary Table 1). The largest functional category among the virulence genes with variable presence were toxins (33% of the variable genes).

#### Toxins

All the hemolysin genes (*hla, hlb, hld*, and *hemolysin III)* and most of the genes coding for leukotoxins (gamma hemolysin and bicomponent leukocidins lukGH, lukED, and/or lukEvDv) were detected in all isolates (Table 3). The genes *lukM* and *lukF,’* coding for bicomponent leukotoxin MF′ were not detected in three isolates, two of which belonged to the peracute group and one to the control group, all CC97. Only the genes *lukS-PV* and *lukF-PV* coding for the bicomponent leukotoxin Panton-Valentine leukocidin remained undetected in any of the isolates.

**TABLE 3:** Distribution of toxin genes in 20 *Staphylococcus aureus* isolates from bovine mastitis milk samples classified by clonal complex (CC) and study group.

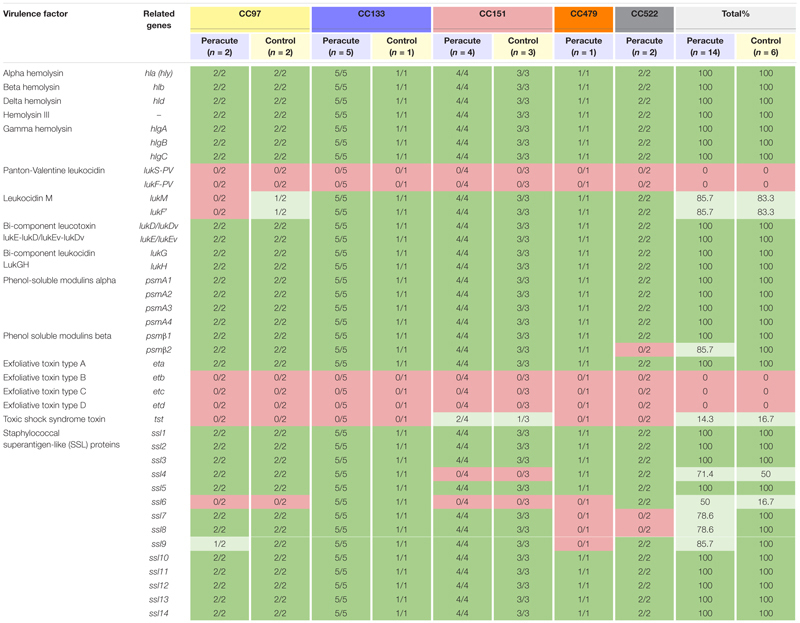

*Darker green color indicates genes present in all isolates within a given group; lighter green indicates genes present in part of the isolates within a given group; red indicates genes not detected in any isolates within a given group.*

Genes coding for phenol-soluble modulins (PSMs) were detected in all isolates, except *psm*β*2*, which was lacking from the two peracute isolates in CC522. Of the exfoliative toxins, the type A gene *eta* was detected in all isolates, but *etb, etc*, and *etd* were detected in none. The *tst* gene coding for toxic shock syndrome toxin was present only in three isolates (1 peracute non-gangrenous, 1 peracute gangrenous, 1 control) belonging to CC151. Some differences were apparent in the presence of the group of genes coding for staphylococcal superantigen-like (SSL) proteins (Table 3).

#### Enterotoxins

The only enterotoxin genes present in all the isolates were those genes coding for the enterotoxin-like proteins W and X. Genes coding for enterotoxins were not commonly found in the isolates (Table 4). Genes of enterotoxin gene cluster 2 were present in all isolates of CC151 and CC479, but absent from all other isolates. All CC151 isolates also carried the enterotoxin-like protein Z and Y genes *selz* and *sey*, and some also the genes coding for enterotoxin L and the bovine variant of enterotoxin C (Table 4).

**TABLE 4:** Distribution of enterotoxin and enterotoxin-like genes in 20 *Staphylococcus aureus* isolates from bovine mastitis milk samples classified by clonal complex (CC) and study group.

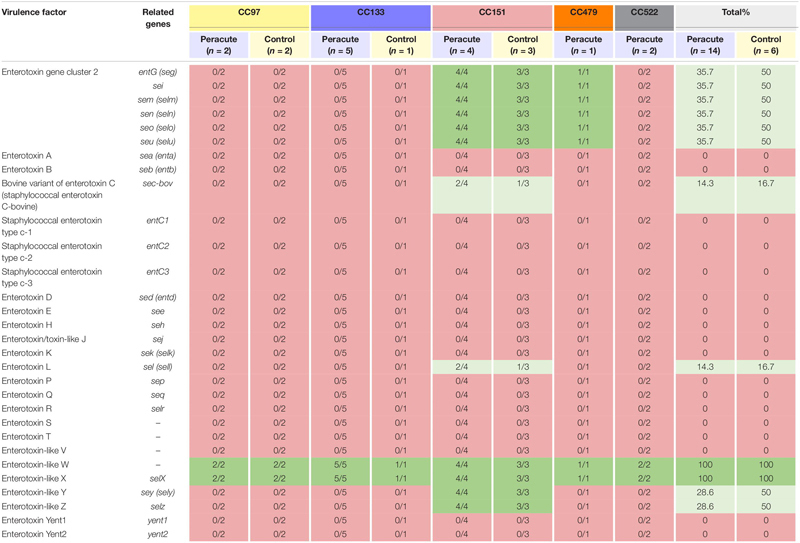

*Darker green color indicates genes present in all isolates within a given group; lighter green indicates genes present in part of the isolates within a given group; red indicates genes not detected in any isolates within a given group.*

#### Host Immune Evasion

The genes required for capsule formation were present in all isolates. The isolates in CC97 belonged to capsular type 5, and all other isolates to capsular type 8 (Table 5). None of the isolates carried *chp* coding for chemotaxis inhibitory protein of *Staphylococcus aureus* (CHIPS), but all isolates carried seven other host immune evasion-related genes (Table 5). All isolates carried the staphylococcal complement inhibitor gene *scn*, and in addition, all the isolates in CC133 and CC522, and most (3/4) of the isolates in CC97 also carried the ruminant-specific variant of the gene *scn*.

**TABLE 5:** Distribution of immune evasion genes in 20 *Staphylococcus aureus* isolates from bovine mastitis milk samples classified by clonal complex (CC) and study group.

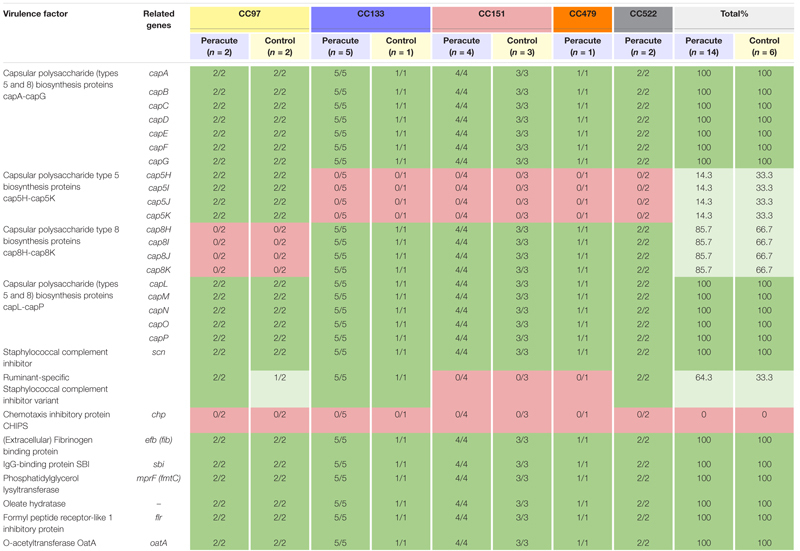

*Darker green color indicates genes present in all isolates within a given group; lighter green indicates genes present in part of the isolates within a given group; red indicates genes not detected in any isolates within a given group.*

#### Iron Uptake and Metabolism

Of the 36 genes associated with iron uptake and metabolism, all except the gene *fhuD1* were present in all isolates. The gene *fhuD1* was detected only in isolates in CC97 (Table 6).

**TABLE 6:** Distribution of exoenzyme genes associated with iron uptake and metabolism in 20 *Staphylococcus aureus* isolates from bovine mastitis milk samples classified by clonal complex (CC) and study group.

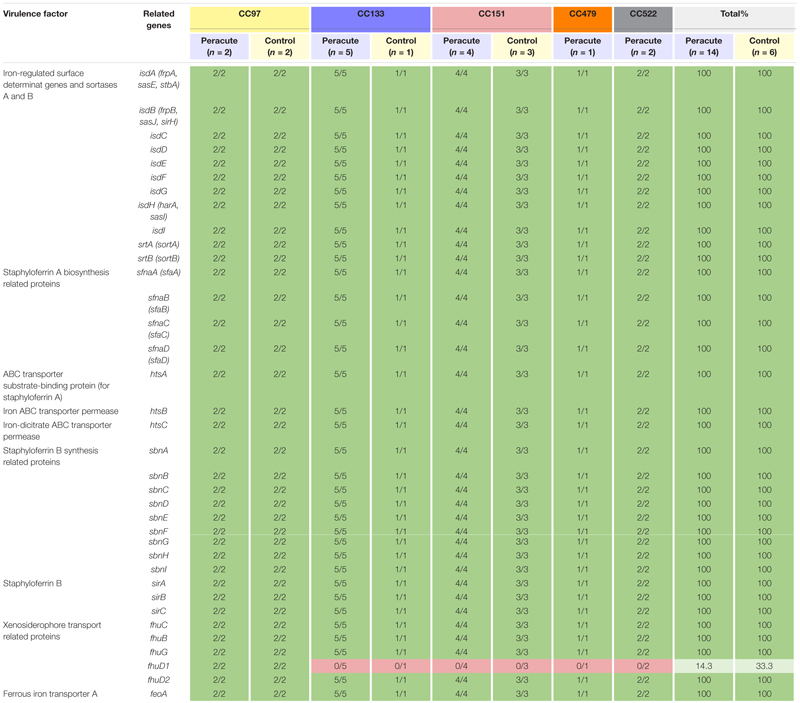

*Darker green color indicates genes present in all isolates within a given group; lighter green indicates genes present in part of the isolates within a given group; red indicates genes not detected in any isolates within a given group.*

#### Exoenzymes

Differences were apparent between isolates in the presence of the serine protease genes *splD, splE, and splF* (Table 7). The isolates in CC522 carried all the genes, the CC479 isolate all but *splF* and all isolates in CC151 all but *splD*. All isolates in CC133 carried *splF* but lacked *splD* and *splE*. All CC97 isolates carried *splD*, but *splE* and *splF* were carried by only two of the four isolates (Supplementary Table 1). All isolates carried the remaining genes in this subcategory, except the gene for staphylokinase, which was not detected in any of the isolates (Table 7).

**TABLE 7:** Distribution of exoenzyme genes (other than iron metabolism) in 20 *Staphylococcus aureus* isolates from bovine mastitis milk samples classified by clonal complex (CC) and study group.

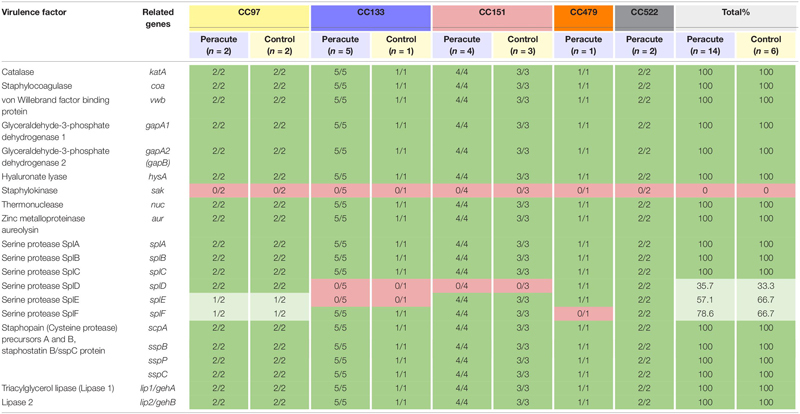

*Darker green color indicates genes present in all isolates within a given group; lighter green indicates genes present in part of the isolates within a given group; red indicates genes not detected in any isolates within a given group.*

#### Adherence-Related Genes

Most adherence-related genes were carried by all isolates (Table 8). The fibronectin-binding protein gene *fnbA* was carried by all isolates, but *fnbB* only by all isolates in CC97, CC133, and CC479. The gene *cna* coding for collagen-binding adhesion protein was detected only in isolates in CC479 and CC522. Of the genes coding for cell-wall-anchored proteins, *sasB* was not detected in any isolate, but *sasC, sasF*, and *sasH* were detected in all isolates. The *sasD* gene was present in isolates in CC97 and CC151, and *sasG* in isolates in CC97, CC479, and CC522. The *sasK* gene was present in isolates in CC97, CC133, CC479, and CC522. The *bap* gene coding for the surface protein involved in biofilm formation was not detected in any of the isolates. The gene *sdrD* was present in isolates in CC133 and CC522, and *sdrE* in isolates in CC133, CC479, CC522, and in all but one isolate in CC151. In addition, *sdrD* was detected in one isolate and *sdrE* in another isolate in CC97 (Table 8).

**TABLE 8:** Distribution of adherence-related virulence genes in 20 *Staphylococcus aureus* isolates from bovine mastitis milk samples classified by clonal complex (CC) and study group.

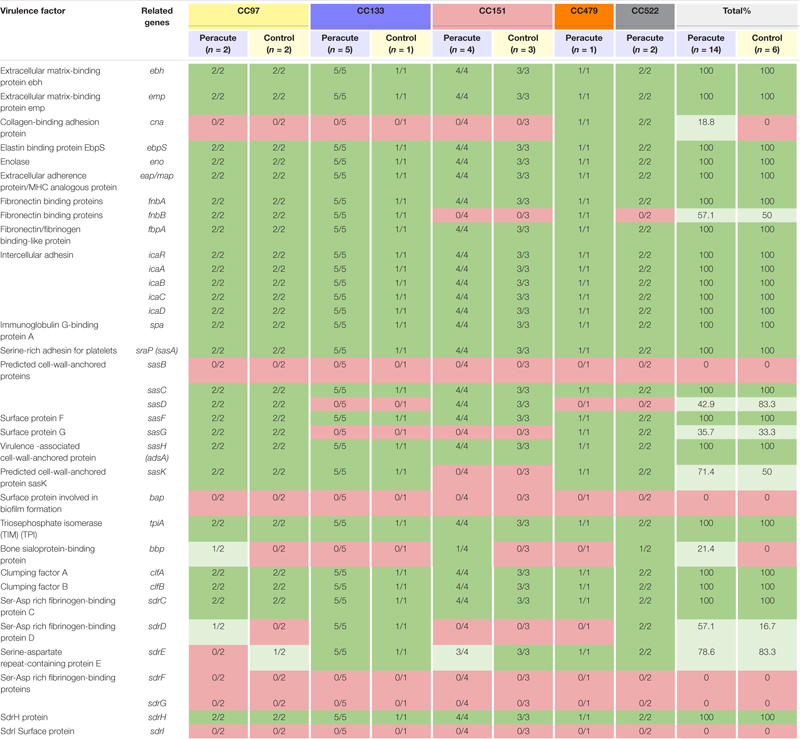

*Darker green color indicates genes present in all isolates within a given group; lighter green indicates genes present in part of the isolates within a given group; red indicates genes not detected in any isolates within a given group. MHC = major histocompatibility complex*

#### Regulatory Genes

Our isolates belong to Agr classes I (12 isolates belonging to CC97, CC133, and CC522) and II (8 isolates belonging to CC151 and CC479). Of the other regulatory genes, *sarT* and *sarU* were lacking in isolates in CC133 and CC151, and *sarB* and *sarD* in all isolates. All the remaining 40 regulatory genes were present in all isolates (Supplementary Table 1). The *rot* gene coding for helix-turn-helix (HTH)-type transcriptional regulator was present in all isolates. However, variations in *rot* gene sequences existed between the isolates. Four *rot* gene variations were apparent, of which one was most common, and were shared by 15 isolates belonging to CC97, CC133, CC151, and CC522 (Table 9). Two isolates of CC97 shared one *rot* gene variant, as well as two isolates in CC151. The single isolate in CC479 carried its own *rot* variant. A functional *rot* gene was missing from three isolates, due to lack of a proper start codon and because of a point mutation at position 2 (atg → aag). These were the isolates in CC479 and CC522.

**TABLE 9:** Distribution of regulatory genes in 20 *Staphylococcus aureus* isolates from bovine mastitis milk samples classified by clonal complex (CC) and study group.

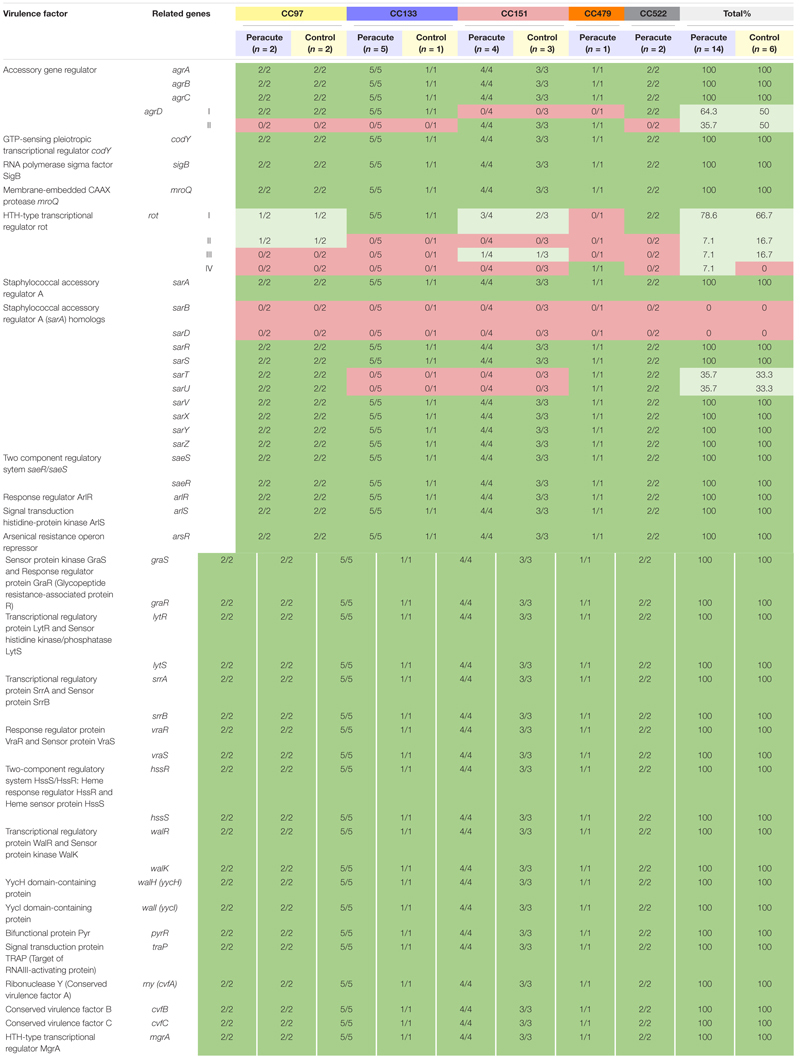

*Darker green color indicates genes present in all isolates within a given group; lighter green indicates genes present in part of the isolates within a given group; red indicates genes not detected in any isolates within a given group.*

*GTP, guanosine triphosphate; HTH, helix-turn-helix; CAAX, cysteine-2 aliphatic amino acids-terminal amino acid.*

#### Secretion Systems and Miscellaneous Virulence-Related Genes

All the 21 genes in the category Secretion systems were present in all the isolates (Supplementary Table 1). Four genes (*pls, edin, edinB, edinC*) in the category Miscellaneous were not detected in any of the isolates. All the other 37 genes in this category were present in all the isolates (Supplementary Table 1).

### Antimicrobial Resistance Genes

The genes responsible for resistance to various antimicrobials were not commonly found in our isolates. Based on the RGI analysis, none of the isolates carried the *blaZ*, *mecA*, or *mecC* genes responsible for beta-lactam resistance. Eight antimicrobial resistance (AMR)-associated genes were detected in all isolates: *mepA* (multidrug export protein mepA) and its repressor *mepR*, *norA* (quinolone resistance protein norA) and its regulators *arlS* (signal transduction histidine-protein kinase ArlS) and *arlR* (response regulator ArlR), *tet*(38) (tetracycline efflux MFS transporter), *LmrS* (major facilitator superfamily multidrug efflux pump), and *mgrA* (HTH-type transcriptional regulator MgrA, also known as NorR), which is a positive regulator for norA expression and repressor for norB and tet38. In addition, isolates other than CC97 carried from two to three more AMR genes. *murA* (antibiotic-resistant murA transferase) was detected in CC133, CC151, CC479, and CC522, *glpT* (antibiotic-resistant GlpT) in most of the CC133 and all CC151, CC479, and CC522 isolates, and *fosB* (fosfomycin thiol transferase) only in CC133 isolates (Supplementary Table 5).

### Association Between Presence of Virulence Genes or Clonal Complex and Type of Mastitis

In general, there was little variation in the presence of the virulence genes between the isolates belonging to the peracute or control groups. The isolates in each CC showed highly similar virulence gene profiles. Of the 296 VFs studied, 42 were variably present. The variation in the presence of these 42 genes existed mainly between the CCs. In 12 of these genes, variation in presence existed even between isolates in the same CC: nine genes in CC97, five genes in CC151, and one gene in CC522 varied between the isolates. CC133 was overrepresented in the peracute group: 5 of 6 CC133 isolates that originated from peracute mastitis (4 from peracute gangrenous mastitis), and 36% of our isolates from peracute mastitis (44% of isolates from peracute gangrenous mastitis) belonged to CC133. The genes *lukM* and *lukF′*, both carried by the same isolates, were the only genes that showed a tendency to be associated with peracute gangrenous mastitis in Fisher’s exact test (*p* = 0.08). The *lukM* and *lukF′* genes were present in all 9 isolates of the peracute gangrenous group, 3 of 5 isolates of the peracute non-gangrenous group, and 5 of 6 control isolates (Supplementary Table 1). The presence of the *sasD* gene was significantly associated (*p* = 0.04) with the control group (5 of 6 isolates) and the peracute non-gangrenous group (4 of 5 isolates). In the peracute gangrenous group, only 2 of 9 isolates carried *sasD* (Supplementary Table 1). Similarly, the presence of the *fhuD1* gene (*p* = 0.11) and the capsule type 5 (*p* = 0.11), present only in all CC97 isolates, tended to be associated with the control and the peracute non-gangrenous groups (Supplementary Table 1). In the multivariate mixed-effect logistic regression model, the two composed CC groups (1 = CC97 + CC151, 2 = CC133 + CC479 + CC522) were associated with the type of mastitis (control group/peracute non-gangrenous/peracute gangrenous). CC group 2 increased the odds for peracute gangrenous mastitis for cows in parity ≥ 2 (odds ratio 2.1; *p* = 0.018). In CC group 2, 7 of 9 isolates were from peracute gangrenous mastitis, compared with CC group 1, in which 2 of 11 isolates originated from peracute gangrenous mastitis (Supplementary Table 1). Parity of the cow was not significant overall (*p* = 0.09, Wald test), but the odds ratio for having gangrenous mastitis was higher for parity 3–4 than for parity 1–2 cows (odds ratio 34.6; *p* = 0.03). Without merging the CCs into the two previously mentioned groups, the association between the CCs and type of mastitis was not detected (*p* = 0.47, Wald test in univariable analysis), probably because of the low number of isolates in the various CCs.

## Discussion

To our knowledge, no previous studies have performed a comparative genomic analysis focusing on *S. aureus* isolates from peracute bovine mastitis. This is most likely because peracute *S. aureus* mastitis cases occur rarely and thus collecting the isolates is time-consuming. The rareness of this *S. aureus* mastitis type also affected our sample size. Even though we included all peracute gangrenous or non-gangrenous *S. aureus* mastitis cases from 2011 to 2018 in the practice area of The Ambulatory Clinic of the Faculty of Veterinary Medicine, University of Helsinki, our sample size is limited. However, the small sample size enabled us to report the clinical signs and the case outcomes in exceptional detail. We think our study provides valuable information on the genomic properties of *S. aureus* isolates from these rare cases. However, more field *S. aureus* isolates from peracute bovine mastitis will be required in future analysis.

Several studies have focused on the possible differences in virulence gene profiles between *S. aureus* isolates from clinical and subclinical mastitis, but have failed to detect any differences (Åvall-Jääskeläinen et al., 2018; Rocha et al., 2019; Naushad et al., 2020). Mastitis is a dynamic phenomenon; when the microbe enters the mammary gland, the leukocytes in the milk and mammary-gland epithelial cells react and initiate the immunologic defense process, leading to large numbers of neutrophils migrating to the mammary gland and attempting to kill the microbial cells (Wellnitz and Bruckmaier, 2012). This phase is often accompanied by clinical signs of varying magnitude. However, if the immune system fails to destroy all the microbes, as typically happens in *S. aureus* infection, the infection persists and continues as subclinical, without clinical signs but with milk somatic cell count (SCC) above the normal level (Rainard et al., 2018a; Coté-Gravel and Malouin, 2019). Our aim here was to focus on the most severe cases of *S. aureus* mastitis; if any specific virulence genes or non-classical virulence determinants/markers are responsible for the severe course of mastitis, these genes should be more prevalent in isolates from mastitis with very severe symptoms than in isolates from subclinical or clinical mastitis with less severe symptoms.

Despite our small data set, we performed statistical analyses to discover any associations between virulence genes and the type of mastitis. When the presence of virulence genes throughout the group of 14 isolates from peracute mastitis was compared with that of the six control group isolates, no statistically significant differences were detected. However, when the peracute group was further divided into nine isolates of gangrenous and five of non-gangrenous mastitis based on the clinical signs, we observed that the genes *lukM* and lukF′, coding for a bicomponent leukotoxin, tended (*p* = 0.08) to be more common in isolates originating from gangrenous mastitis than in isolates from non-gangrenous mastitis and the control group. Several studies have found LukMF′ to be the most potent toxin in killing bovine neutrophils *in vitro* (Barrio et al., 2006; Vrieling et al., 2016), and it is the leucocidin most abundantly secreted *in vitro* by *S. aureus* mastitis isolates (Vrieling et al., 2016). The gene *sasD* coding for cell-wall-anchored protein D was statistically significantly associated with the non-gangrenous mastitis and control groups (*p* = 0.04) in our study. However, the *lukM* and *lukF′* genes were lacking only from three of the four CC97 isolates, and *sasD* was present only in CC97 and CC151 isolates. In general, the virulence gene profiles corresponded to CCs, not to the study groups. None of the CCs alone was statistically significantly associated with any of the symptom groups in our study (control, peracute non-gangrenous, peracute gangrenous), but when the CCs were grouped into two groups (1 = CC97 + CC151, 2 = CC133 + CC479 + CC522), the gangrenous group was statistically significantly associated with CC group 2. Grouping of CCs was necessary because of the small amount of data.

The majority, 17 of 20 (85%) of our isolates belonged to three CCs: CC151, CC133, and CC97, which have been reported as common bovine-associated complexes (Hoekstra et al., 2020; Naushad et al., 2020). Two of our isolates in the peracute group belonged to CC522, which is commonly found in isolates from mastitis in small ruminants (Kahila Bar-Gal et al., 2015; Romano et al., 2020). A recent review (Matuszewska et al., 2020) showed that CC522 has thus far been found only in small ruminants. In a recent study analyzing 276 *S. aureus* isolates from clinical and subclinical bovine mastitis from 11 European countries (Hoekstra et al., 2020), 58% of the isolates belonged to CC151, CC133, and CC97, of which CC133 was the 4th most common (9.1%) in that study. The 3rd most common isolate in that study, CC479, represented 11.6% of the isolates, while only one isolate (5%) in our study belonged to CC479. In another recent whole-genome sequencing study analyzing 119 *S. aureus* isolates from clinical and subclinical bovine mastitis from Canada (Naushad et al., 2020), CC151 and CC97 were the most common, covering 92% of the isolates. Only one isolate in that study belonged to CC133, while CC479 and CC522 were not detected at all. We found that CC133 was overrepresented in the peracute mastitis group, since more than one third (36%) of our isolates in the peracute mastitis group belonged to CC133. In the peracute gangrenous subgroup, 44% of the isolates belonged to CC133. Interestingly, the *S. aureus* isolates of CC133 induced a significantly stronger interleukin 8 (IL-8) release from bovine mammary epithelial cells in cell culture than isolates from the other CCs (Hoekstra et al., 2019). In the 11 European countries that participated in the study by Hoekstra et al. (2020), the proportion of CC133 in each country varied from 0 to 25%. Finland was not included in these countries. Hoekstra et al. (2020) found no association between this CC and clinical mastitis, although they did find an association between clinical mastitis and CC479. The single CC479 isolate in our study originated from peracute gangrenous mastitis and clustered in ribotyping with the two isolates belonging to CC522, also from peracute gangrenous mastitis. CC522 was not detected by Hoekstra et al. (2020), nor by Naushad et al. (2020). Both CC522 and CC133 are commonly found in small ruminants (Kahila Bar-Gal et al., 2015; Romano et al., 2020). Neither of the herds from which the CC522 isolates in our study originate has any association with small ruminants, nor are these farms located adjacent to each other.

The normal metabolic capabilities of *S. aureus* can also be linked with its pathogenicity (Bosi et al., 2016). In the study of Rocha et al. (2019), certain COG families that contain metabolic determinants not directly or classically linked with virulence (e.g., potassium metabolism, cofactors, and vitamins), were overrepresented in one clinical mastitis strain in comparison to two subclinical mastitis strains and one clinical strain. Such differences could not be detected between our study groups, because all the various COG families were similarly distributed in the peracute and control groups. In the GO enrichment analysis, which was performed on annotated genes found only in peracute isolates, some GO terms, including those that are not directly associated with classical VFs (e.g., transporter activity), were significantly enriched. Further analysis would, however, be needed to examine more specifically which genes are included in these GO terms. Our results thus far suggest that even some non-classical *S. aureus* virulence determinants could be of relevance in the clinical outcome of peracute mastitis with very severe signs.

At least some allelic variation was present in virulence genes both between and within the CCs. We focused only on the allelic variation in the *rot* gene coding for a transcriptional regulator, repressor of toxins, which may be involved in LukMF′ regulation (Hoekstra et al., 2018). *Staphylococcus aureus* isolates from bovine mastitis carrying the *LukM-lukF′* genes produce varying amounts of LukMF′ protein both *in vitro* and *in vivo*, while high LukMF′ production has been associated with severe signs of mastitis (Vrieling et al., 2016; Hoekstra et al., 2018). Hoekstra et al. (2018) found that isolates belonging to CC479 and *spa*-type t543 showed 10-fold higher *in vitro* LukMF′ production than the average of the other isolates from 38 clinical and 17 subclinical mastitis cases from 33 different farms. Two point mutations in the *rot* gene were found in these LukMF′ high-producing isolates, of which the one at position 2 caused the loss of the start codon and thus a non-functional *rot* gene (Hoekstra et al., 2018). Interestingly, three isolates, belonging to CC479 and CC522, all originating from peracute gangrenous mastitis cases, shared a point mutation in position 2 in the *rot* gene and thus no start codon. In total, four rot gene sequence variants were detected among our isolates, of which one predominated (15 of 20 isolates). This variation was, however, not associated with the type of mastitis.

Peracute severe mastitis and especially peracute gangrenous *S. aureus* mastitis occur rarely, but frequently enough in dairy cows (Matsunaga et al., 1993; Shibahara and Nakamura, 1999; Shafi et al., 2015), although the vast majority of *S. aureus* mastitis cases are subclinical or clinical with mild to moderate symptoms and tend to develop persistence despite appropriate antimicrobial therapy (Barkema et al., 2006). Histopathological analysis of severe necrotizing *S. aureus* mastitis is characterized by vascular necrosis with fibrinous thrombosis, necrosis of the interlobular and intralobular ducts and alveolar epithelial cells (Shibahara and Nakamura, 1999). Some authors have suggested that the severity of mastitis is due to the host immune response (Rainard et al., 2018b). Mastitis caused by *S. aureus* may develop into gangrenous mastitis if bacteria are allowed, through lack of bacterial killing by neutrophils in milk, to multiply without inhibition. Schalm et al. (1976) caused neutropenia in four study cows with chronic *S. aureus* mastitis induced earlier with an alpha-, beta- and delta-toxic *S. aureus* strain, by administering equine anti-bovine leukocyte serum by continuous intravascular drip. Neutropenia in blood was reflected as a reduction in polymorphonuclear leukocytes and an increase in *S. aureus* numbers in milk in two cows, of which one developed gangrenous mastitis and the other died when gangrenous mastitis was in the initial stages of development. The remaining two cows were able to maintain high milk leukocyte levels despite decrease in blood leukocyte level and did not develop gangrenous mastitis. However, Rainard et al. (2018b) showed in an experiment using goats that the lack of phagocytic efficiency in the milk was not due to insufficient cell concentration. In this study, 10 goats of two different breeding lineages were challenged with two different *S. aureus* strains. Five goats developed severe mastitis symptoms, including four with gangrenous mastitis, while five other goats developed mild to moderate symptoms. No relationship between the severity of mastitis and goat breeding lineage or *S. aureus* strain was detected.

Although the host response to intramammary infection may play a crucial role in the development of mastitis symptoms, there are indications that bacterial properties do have an impact as well. For example, Haveri et al. (2005) showed that a group of genetically related *S. aureus* isolates was associated with severe clinical mastitis. They pulsotyped *S. aureus* isolates from 134 mastitic bovine mammary quarters. While the other most prevalent pulsotypes included isolates mainly from mild clinical or subclinical mastitis, more than half of the isolates in one pulsotype originated from severe clinical mastitis. Also, some experimental mastitis studies have demonstrated indications of differences in *Staphylococcus* species or strains affecting the intensity of inflammation (Piccart et al., 2016; Vrieling et al., 2016; Niedziela et al., 2020).

Differences in protein production of *S. aureus* isolates from different types of mastitis have also been detected *in vivo* using seroproteomics. Le Maréchal et al. (2011) challenged the mammary glands of 12 ewes with two different but genetically similar *S. aureus* strains, of which one was isolated from ovine subclinical mastitis and the other from ovine gangrenous mastitis in the same herd (Vautor et al., 2009). The authors found that five of six ewes challenged with the strain originating from gangrenous mastitis developed gangrenous mastitis, but only one of the six ewes challenged with the strain from subclinical mastitis did so. Twelve immunogenic staphylococcal proteins were specific for the gangrenous mastitis strain. However, the genes coding for these 12 proteins were present and highly similar in both strains, indicating that reasons other than the simple presence of the genes cause the strain isolated from gangrenous mastitis to produce these proteins *in vivo* during mastitis but not, or to a much lesser extent, the strain isolated from subclinical mastitis (Le Maréchal et al., 2011). The answer to the question of how the intensity of inflammation and the severity of clinical signs of mastitis is determined will not be found simply by examining the presence of virulence genes, nor solely the immunological defense capabilities of the host, but possibly in the allelic variation in the genes and especially the factors that moderate their expression.

To summarize, very little variation was detected in the presence of virulence genes between the isolates. Most (74%) of the VFs were detected among all isolates.

Five clonal complexes, CC79, CC133, CC151, CC479, and CC522 were detected in our study. To our knowledge, CC522 has not been previously reported in bovine mastitis. The other four CCs detected in our study have also been among the predominant CCs in earlier bovine *S. aureus* mastitis studies (Hoekstra et al., 2020; Naushad et al., 2020). Three CCs, namely CC133, CC479, and CC522, were significantly associated with peracute gangrenous *S. aureus* mastitis.

Even though the immunological capabilities of the host most likely play a significant role in determining the clinical outcome, pathogen-related factors such as VFs also have an impact. However, the clinical signs of *S. aureus* mastitis are probably not associated with the mere presence of any virulence genes but may be affected by allelic variation and especially different regulation and thus expression in the virulence genes.

## Data Availability Statement

All whole-genome sequencing data used in this study are available from NCBI (https://www.ncbi.nlm.nih.gov) under BioProject ID PRJNA590109.

## Author Contributions

SÅ-J, JK, HS, and ST conceived, designed the experiments, authored and reviewed drafts of the manuscript, and approved the final draft. SÅ-J, JK, and ST performed the bioinformatics analysis and analyzed the data. HS organized sample collection from the farms and performed the statistical analysis. All authors contributed to the article and approved the submitted version.

## Conflict of Interest

The authors declare that the research was conducted in the absence of any commercial or financial relationships that could be construed as a potential conflict of interest.

## Footnotes

1 https://pubmlst.org/saureus

2 https://cge.cbs.dtu.dk/services/spatyper

3 http://www.uniprot.org

4 https://aureowiki.med.uni-greifswald.de/Main_Page

5 https://card.mcmaster.ca/home

## Supplementary Material

Supplementary Figure 1 Pangenome characterization based on distribution of functional categories. Distribution of pangenome into functional categories obtained after comparing the pangenome with the Clusters of Orthologous Groups (COG) reference database. The average amount of proteins in each COG category is shown for the peracute group of *Staphylococcus aureus* isolates (red bars) and control group of *S. aureus* isolates (gray bars). Standard deviation between isolates is indicated in each bar.
