# Supplementary figures and images for: Genomic Analysis of Staphylococcus aureus Isolates Associated With Peracute Non-gangrenous or Gangrenous Mastitis and Comparison With Other Mastitis-Associated Staphylococcus aureus Isolates
Source: Front Microbiol. 2021 Jul 8;12:688819. doi: 10.3389/fmicb.2021.688819 (PMC8297832; doi:10.3389/fmicb.2021.688819)

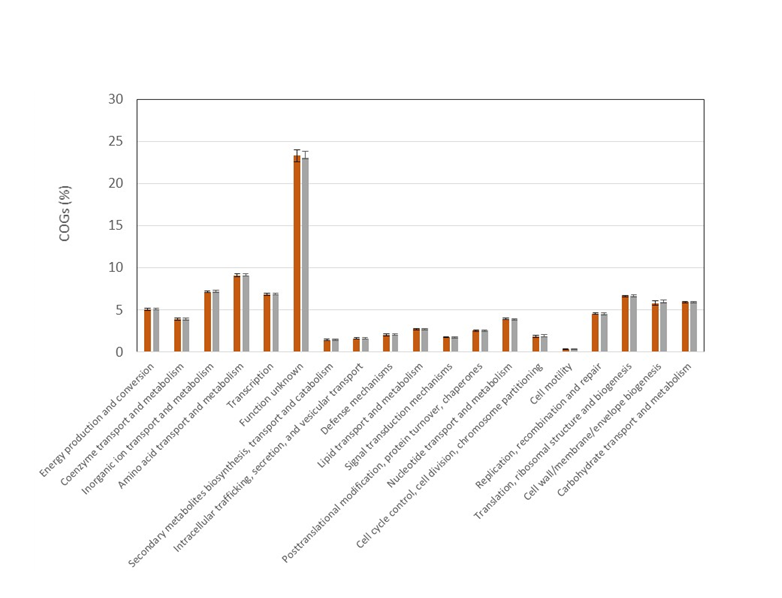

Supplement: Supplementary Figure 1 — Pangenome characterization based on distribution of functional categories. Distribution of pangenome into functional categories obtained after comparing the pangenome with the Clusters of Orthologous Groups (COG) reference database. The average amount of proteins in each COG category is shown for the peracute group of Staphylococcus aureus isolates (red bars) and control group of S. aureus isolates (gray bars). Standard deviation between isolates is indicated in each bar. [file Image_1.TIF]
