# Supplementary material for: Genomic Analysis of Staphylococcus aureus Isolates Associated With Peracute Non-gangrenous or Gangrenous Mastitis and Comparison With Other Mastitis-Associated Staphylococcus aureus Isolates
Source: Front Microbiol. 2021 Jul 8;12:688819. doi: 10.3389/fmicb.2021.688819 (PMC8297832; doi:10.3389/fmicb.2021.688819)
Supplement: Supplementary file 3 [file Table_2.DOCX]

Supplementary Table S2. General properties of *Staphylococcus* draft genome sequences used in this study.

| Isolate | \| **Contigs (N)** \| \| --- \| | Genome length (bp) | GC (%) | Average coverage | BioSample ID |
| --- | --- | --- | --- | --- | --- | --- |
| \| **Saari 1** \| \| --- \| | 43 | 2808570 | 32.74 | 169 | SAMN13319580 |
| Saari 2 | 42 | 2721603 | 32.72 | 110 | SAMN13319581 |
| Saari 3 | 41 | 2712393 | 32.79 | 380 | SAMN13319582 |
| Saari 4 | 48 | 2669120 | 32.65 | 144 | SAMN13319583 |
| Saari 5 | 47 | 2705497 | 32.8 | 86 | SAMN13319584 |
| Saari 6 | 38 | 2789452 | 32.79 | 110 | SAMN13319585 |
| Saari 7 | 37 | 2654962 | 32.65 | 110 | SAMN13319586 |
| Saari 8 | 39 | 2789283 | 32.79 | 153 | SAMN13319587 |
| Saari 9 | 42 | 2658696 | 32.65 | 121 | SAMN13319588 |
| Saari 10 | 18 | 2741122 | 32.77 | 113 | SAMN13319589 |
| Saari 11 | 45 | 2706022 | 32.8 | 73 | SAMN13319590 |
| Saari 12 | 44 | 2709807 | 32.8 | 256 | SAMN13319591 |
| Saari 13 | 42 | 2669301 | 32.65 | 97 | SAMN13319592 |
| Saari 14 | 37 | 2757112 | 32.82 | 78 | SAMN13319593 |
| Saari 15 | 41 | 2663422 | 32.65 | 174 | SAMN13319594 |
| Saari 16 | 37 | 2706261 | 32.8 | 171 | SAMN13319595 |
| Saari 17 | 35 | 2708335 | 32.8 | 215 | SAMN13319596 |
| Saari 18 | 38 | 2708300 | 32.8 | 115 | SAMN13319597 |
| Saari 19 | 39 | 2654707 | 32.65 | 62 | SAMN13319598 |
| Saari 20 | 38 | 2659837 | 32.66 | 147 | SAMN13319599 |

bp = base pairs, GC = guanine-cytosine content
