# Supplementary material for: Genomic Analysis of Staphylococcus aureus Isolates Associated With Peracute Non-gangrenous or Gangrenous Mastitis and Comparison With Other Mastitis-Associated Staphylococcus aureus Isolates
Source: Front Microbiol. 2021 Jul 8;12:688819. doi: 10.3389/fmicb.2021.688819 (PMC8297832; doi:10.3389/fmicb.2021.688819)
Supplement: Supplementary file 4 [file Table_3.DOCX]

Supplementary Table S3. Genome statistics of *Staphylococcus* draft genome sequences used in this study.

| Isolate | CDS^a^ | CRISPR^b^ | Misc RNA^c^ | rRNA^d^ | tmRNA^e^ | tRNA^f^ | Total features |
| --- | --- | --- | --- | --- | --- | --- | --- |
| Saari 1 | 2621 | 1 | 122 | 10 | 1 | 57 | 2812 |
| Saari 2 | 2500 | 0 | 110 | 10 | 1 | 61 | 2682 |
| Saari 3 | 2566 | 1 | 123 | 8 | 1 | 61 | 2760 |
| Saari 4 | 2496 | 0 | 106 | 9 | 1 | 60 | 2672 |
| Saari 5 | 2498 | 1 | 110 | 9 | 1 | 61 | 2680 |
| Saari 6 | 2507 | 1 | 112 | 9 | 1 | 60 | 2690 |
| Saari 7 | 2507 | 0 | 112 | 10 | 1 | 61 | 2691 |
| Saari 8 | 2490 | 0 | 100 | 10 | 1 | 60 | 2661 |
| Saari 9 | 2498 | 1 | 115 | 9 | 1 | 60 | 2684 |
| Saari 10 | 2541 | 0 | 106 | 9 | 1 | 58 | 2715 |
| Saari 11 | 2500 | 1 | 123 | 9 | 1 | 61 | 2695 |
| Saari 12 | 2508 | 0 | 107 | 9 | 1 | 60 | 2685 |
| Saari 13 | 2502 | 0 | 112 | 10 | 1 | 61 | 2686 |
| Saari 14 | 2617 | 1 | 122 | 9 | 1 | 60 | 2810 |
| Saari 15 | 2491 | 0 | 107 | 8 | 1 | 51 | 2658 |
| Saari 16 | 2615 | 1 | 122 | 9 | 1 | 60 | 2808 |
| Saari 17 | 2495 | 0 | 111 | 9 | 1 | 60 | 2676 |
| Saari 18 | 2540 | 1 | 125 | 10 | 1 | 60 | 2737 |
| Saari 19 | 2506 | 0 | 112 | 10 | 1 | 61 | 2690 |
| Saari 20 | 2502 | 0 | 107 | 10 | 1 | 56 | 2676 |

^a^CDS, coding sequence

^b^CRISPR, clustered regularly interspaced short palindromic repeats

^c^Misc RNA, miscellaneous RNA (ncRNA)

^d^rRNA, ribosomal RNA

^e^tmRNA, transfer-messenger RNA

^f^tRNA, transfer RNA
