# Supplementary material for: Genomic Analysis of Staphylococcus aureus Isolates Associated With Peracute Non-gangrenous or Gangrenous Mastitis and Comparison With Other Mastitis-Associated Staphylococcus aureus Isolates
Source: Front Microbiol. 2021 Jul 8;12:688819. doi: 10.3389/fmicb.2021.688819 (PMC8297832; doi:10.3389/fmicb.2021.688819)
Supplement: Supplementary file 5 [file Table_4.DOCX]

Supplementary Table S4. Summary statistics of the pangenome analyses. “All” refers to analysis performed on both peracute and control isolates^1^.

| Group | Definition | All isolates  (n= 20) | Peracute isolates (n=14) | Control isolates  (n= 6) |
| --- | --- | --- | --- | --- |
| Core genes | Present in 99-100% of isolates | 2054 | 2063 | 2120 |
| Soft core genes | Present in 95-99% of isolates | 30 | 0 | 0 |
| Shell genes | Present in 15-95% of isolates | 1040 | 808 | 1125 |
| Cloud genes | Present in 0-15% of isolates | 637 | 846 | 0 |
| Total |  | 3761 | 3717 | 3245 |

^1^The number of predicted protein-coding sequences (CDS) are shown.
